# Deep Learning-Assisted Quality Control of Histology Teaching Slides: Detection and Localization of Tissue Fold Artifacts in H&E-Stained Images

**DOI:** 10.3390/bioengineering13080937

**Published:** 2026-08-19

**Authors:** Osman Fatih Koparir, Berrin Tarakci Gencer, Abdulkadir Sengur

**Affiliations:** 1 Department of Histology Embryology, Faculty of Veterinary Medicine, Firat University, 23100 Elazig, Türkiye; 241304208@firat.edu.tr (O.F.K.); btarakci@firat.edu.tr (B.T.G.); 2 Department of Electrical-Electronics Engineering, Faculty of Technology, Firat University, 23100 Elazig, Türkiye

**Keywords:** tissue fold artifact, hematoxylin-eosin, deep learning, classification, segmentation, quality control

## Abstract

**Background/Objectives**: Tissue fold artifacts observed in hematoxylin-eosin (H&E)- stained preparations used in histology education can complicate the assessment of normal tissue architecture and affect students’ accurate interpretation of microscopic structures. This study aimed to automatically detect and localize tissue fold artifacts in histology teaching preparations using deep learning methods. **Methods**: A total of 2127 hematoxylin and eosin (H&E)-stained histological images of brain, kidney, liver, small intestine, and testis tissues obtained at 10× magnification were used in the study. The dataset consisted of 899 clean/artifact-free images and 1228 images containing tissue fold artifacts. Seven deep learning architectures, including convolutional neural networks (CNN)-based models and a vision transformer-based model, were evaluated for image-level classification: ResNet18, ResNet50, DenseNet121, EfficientNet-B0, EfficientNet-B3, ConvNeXt-Tiny, and Swin-Tiny. Classification performance was evaluated using an organ-based testing approach, a slide-level train–test split in which images from the same histological slide group were retained within a single subset, a general image-level 80/20 train–test split, and five-fold cross-validation. A DeepLabV3-ResNet50-based segmentation model was trained using QuPath-prepared masks to determine fold regions at the pixel level. Grad-CAM was used for qualitative visualization and quantitative comparison with manually annotated fold regions. **Results**: All classification models showed excellent performance overall. The Swin-Tiny model was the most accurate in terms of general image-level classification, scoring 99.06% for accuracy, 99.19% for F1-score, and 99.98% for AUC. In slide-level classification, EfficientNet-B3 showed the best performance in terms of accuracy (99.54%) and AUC (99.99%). No pairwise statistical difference was found among the models using Holm adjustment. In the five-fold cross-validation setting, ResNet50 achieved the best performance with the accuracy of 98.73 ± 0.54%. In the detailed small-intestine error analysis, classification accuracy was 67.67%, with high sensitivity (98.59%) but low specificity (24.34%), mainly because of false-positive predictions. In the segmentation evaluation, the final DeepLabV3-ResNet50 model trained with combined BCE + Dice loss resulted in Dice of 0.7630 ± 0.2425 and IoU of 0.6661 ± 0.2577 on the independent test set. False positive segmentations were rare in 899 artifact-free images (only 0.33% of images had a tissue fold region of at least 1%). The quantitative Grad-CAM analysis showed poor spatial agreement with the manually annotated tissue fold masks (Dice = 0.2423; IoU = 0.1441). In the independent MPP10 dataset, ResNet50 achieved 89.63% accuracy and 88.49% F1-score. **Conclusions**: The suggested method was able to detect tissue fold artifacts as well as localize them in the teaching images of histology. The strong performance recorded at the slide level validates the internal findings, but the limited performance in the case of small intestine images and on the external dataset reveals that tissue structure remains a crucial factor.

## 1. Introduction

In histology courses which are part of medical, dental, veterinary, and health sciences education various theoretical lessons covering different cells, tissues, and organs are taught, along with practical laboratory sessions designed to facilitate a better understanding of the subject [1,2]. Generally, histological microscope slides are commonly employed in practical courses, and the histological preparations utilized in these courses serve as the fundamental teaching materials designed to illustrate tissue structure, cellular structures, and morphological characteristics that vary by organ [3,4].

In particular, H&E stained sections, used in over 80% of histological slides worldwide, are widely used in histology education to help students recognize normal tissue architecture and develop microscopic evaluation skills [5,6]. However, the preparation of hematoxylin eosin stained histological slides used in histology laboratory courses is a multi-stage process involving fixation, tissue processing, embedding, sectioning, staining, and mounting. Technical issues arising at any of these stages can lead to various artifacts that negatively affect the quality of the slides [7,8]. One of the most common technical artifacts in H&E stained histological sections is tissue fold artifacts, which are observed in more than 40% of cases and can distort tissue morphology, thereby complicating diagnosis and the understanding of tissue structure [9]. Tissue fold artifacts generally occur when tissue folds back on itself during microtome sectioning, transfer of the section to a water bath, or placement on a slide. In these areas, tissue thickness increases, cellular structures overlap, and the appearance may differ from the normal histological architecture [10,11]. This difference in histological architecture can negatively impact students’ abilities to identify organs, assess tissue relationships, and understand tissue integrity, particularly in histology education; in other words, it can make it difficult for them to distinguish between normal tissue structures and technical artifacts [12,13,14,15,16].

With the advancement of digital microscopy and image analysis methods, the computer-aided evaluation of histological images has become increasingly valuable in recent years [17,18]. In particular, the automatic analysis of histopathological images using artificial intelligence and deep learning-based models has become increasingly popular [19,20,21]. The analysis of digital histology images encompasses not only classification/diagnosis but also the quantitative analysis of the tissue morphology, cell morphology, and tissue organization. In this context, the histomics strategy allows one to have an organized way to quantify morphological/structural elements in histological images. However, the accuracy of this quantitative analysis depends on the quality of images and the exact demarcation of tissue regions. Therefore, the identification of any artifacts interfering with the normal histological structure, such as tissue folding artifact, before performing the analysis is necessary to ensure the accuracy of the analysis steps [22]. Research in the field of digital pathology indicates that artifacts not only hinder pathologists’ diagnostic evaluations but can also negatively impact the performance of AI-based diagnostic and classification models by degrading the quality of histological images [23,24,25]. Because of this, AI-based quality control and artifact detection have significantly advanced to address the challenges posed by artifacts such as air bubbles, tissue folds, dust, out-of-focus areas, and pen marks that can compromise diagnostic accuracy and the performance of AI models [26,27].

In recent years, tissue folds have been considered an important class of artifacts in artificial intelligence-based systems developed for quality control of histopathological specimens in digital pathology [28,29]. These systems are aimed at detecting and masking areas containing tissue fold artifacts in whole-slide images, or at accelerating the quality assessment process [30]. GrandQC, HistoQC, and similar approaches are important examples of the automation of quality control in histological images [27,31]. However, the majority of current studies focus on whole-slide images obtained from diagnostic pathology archives; studies on the evaluation of artifacts in student specimens used in histology education using artificial intelligence remain limited [24,32,33].

This study aimed to develop a deep learning-assisted approach for quality control in histology teaching slides by detecting and localizing tissue fold artifacts in H&E-stained images. The image set included 10× magnification samples from five organs commonly used in histology practical courses: liver, brain, testis, small intestine, and kidney. The study was based on the premise that tissue fold artifacts in educational histology specimens can be distinguished from clean/artifact-free tissue areas through deep learning-based image analysis. The experimental design included both classification and segmentation tasks. In the organ-based classification stage, each organ was used as an independent test set in turn, while the remaining organs were used for training. This allowed the generalization of the models across different tissue types to be examined. A second classification experiment was conducted by combining all organ images and splitting the dataset into 80% training and 20% testing subsets. The reliability of the classification results was also evaluated through five-fold cross-validation. In addition to image-based evaluations, tissue fold artifact localization was performed by means of semantic segmentation, where masks for folds were created manually. The quality of classification was assessed by accuracy, F1 score, ROC-AUC, and loss values, whereas semantic segmentation evaluation was done using Dice and IoU scores. Grad-CAM images were created for visualization of image parts responsible for classification decision. With the help of this combination of methods, this study performed tissue fold artifact detection from multiple angles, such as organ-based generalization, classification results, cross-validation stability, pixel-wise localization, and visual explanation. These results could be potentially applied in the preliminary screening of histology teaching slides and identification of specimens with tissue fold artifacts.

## 2. Materials and Methods

**Figure 1:**
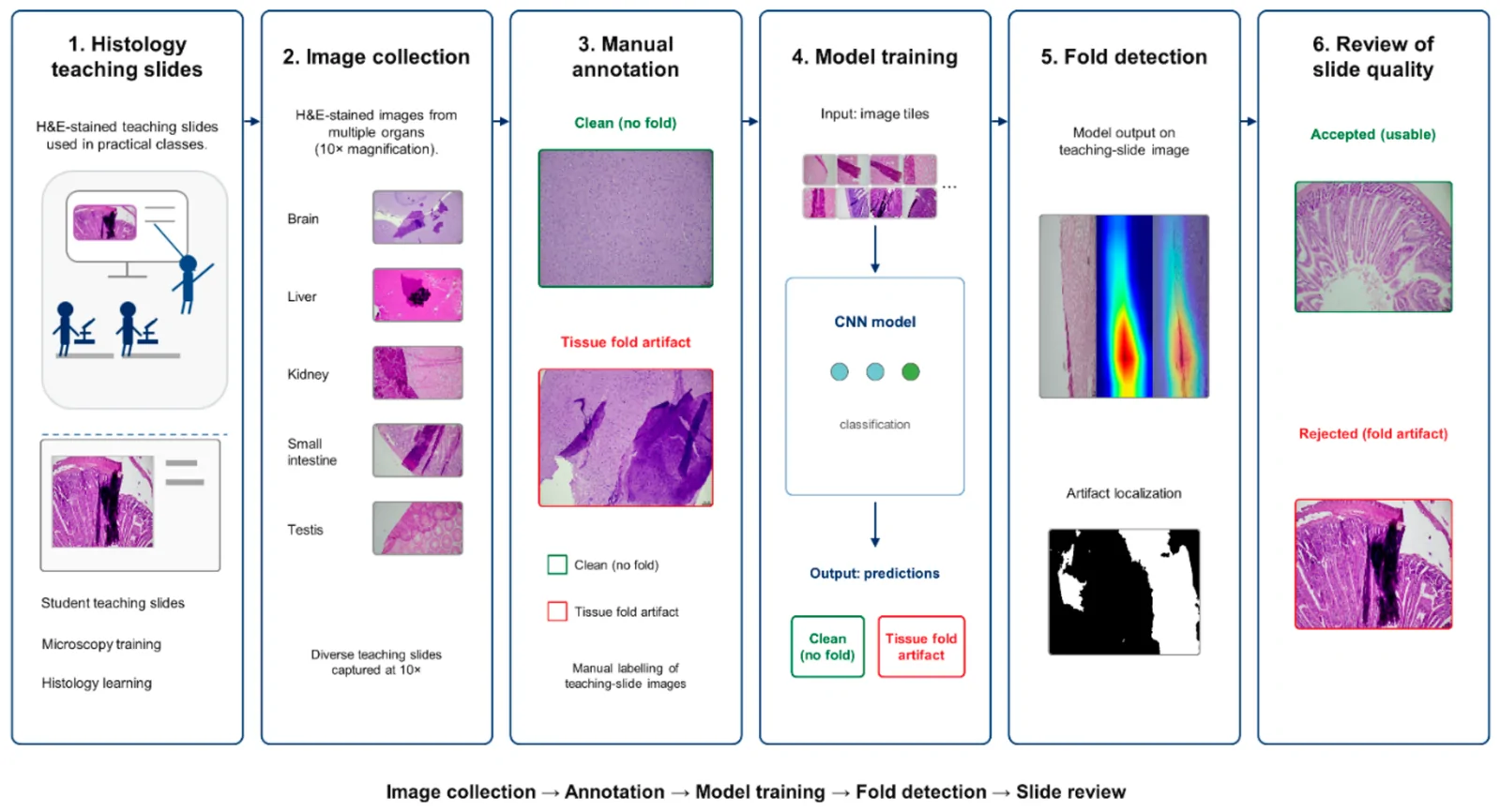
Illustration of the proposed model.

A two-stage deep learning workflow was used to identify tissue fold artifacts. First, images were classified as Clean or Fold; second, fold regions were localized at the pixel level in images containing folds. The models learned image features directly, allowing irregular fold patterns across different organ tissues to be represented without hand-crafted descriptors [34,35,36]. Figure 1 illustrates the workflow.

### 2.1. Deep Learning-Based Image Analysis

CNNs were used for image classification and segmentation [37], learning local image patterns through convolutional operations [38]. ReLU activations and softmax class probabilities were used in the classification models [39,40].

The evaluated residual, dense, EfficientNet, ConvNeXt, and Swin Transformer architectures differed in their feature-learning and information-flow mechanisms [41,42].

### 2.2. Image-Level and Slide-Level Classification of Tissue Fold Artifacts

Image-level classification was defined as a two-class Clean/Fold problem, where Clean images lacked tissue folds while Fold images showed fold-induced changes in morphology. For each of the models, scores for Clean and Fold classes were generated; probabilities were calculated using the softmax activation, and the class with the highest probability was selected [43,44]. Cross-entropy loss function was used to train the image-level classifiers [44]. The seven reference architectures were evaluated under the same experimental conditions to enable direct comparison. UNI was evaluated separately as a frozen feature extractor using the same slide-level train–test partition [45,46].

To reduce information leakage between histological field counterparts, all images belonging to a particular histological slide set were exclusively assigned to either the training set or the test set. Such slide-level splitting was evaluated independently from image-level 80/20 random division and five-fold cross-validation. For the small intestine, which showed the poorest leave-one-organ-out performance, the number of false positives and false negatives from the confusion matrix was calculated, and representative images were evaluated to find common histological patterns causing misclassification.

### 2.3. Learning Representation Based on ResNet

ResNet model was used in the classification stage of the proposed study [47]. Its residual connections reduce the optimization challenges in deep networks [48]. For the target mapping H(x), the residual function was defined as:

F(x)=H(x)−x

The corresponding block output was similarly defined as:

H(x)=F(x)+x

where x is the input feature map, F(x) is the learned residual transformation, and H(x) shows the block output. The shortcut permits identity information to pass through the block [49].

For histological images, the ResNet feature extractor learned color, edge, and texture representations associated with irregular fold patterns [39,48], which were mapped to the Clean or Fold class by the final classification layer.

### 2.4. Transfer Learning and Fine Tuning

Transfer learning was used because of the limited number of labeled histopathology images available [50]. The networks were first pretrained with some weight [51], and their classification layer was customized for the binary problem. Transfer learning of lower-level visual features can aid convergence with histopathology images [52].

As an additional comparison, the UNI pathology foundation model was used as the feature extractor for Clean/Fold [53]. The pre-trained UNI encoder was fixed and showed its ability to produce features at the image level without any further training. Features were standardized based on statistics computed from the training dataset, and then used to train a logistic regression model. The same slide-level training/testing partitioning as that of the reference models was done for consistent data comparisons. Test data were not used in either the feature standardization process or the training of the classifier.

### 2.5. Image Preprocessing

The input images in the histological dataset were resized to 224 × 224, and the images in the segmentation dataset were resized to 512 × 512 for convenience with regards to input in the deep learning models. RGB images were converted to tensors and later normalized with the mean and standard deviation values of the channels corresponding to the pretrained model:

$$x^{\prime }=\frac{x-\mu }{\sigma }$$

where x is the original channel value, μ the channel mean, σ the channel standard deviation, and $x^{\prime }$ the normalized value [54]. Training augmentation included horizontal and vertical flipping, limited rotation, and color changes [55]:

$$\tilde{x}=T\left(x\right)$$

where T(⋅) is the random transformation and $\tilde{x}$ the augmented image.

### 2.6. Pixel-Level Segmentation of the Tissue Fold Region

A semantic segmentation model was used in the second stage to localize fold regions [56], with each pixel assigned to background or fold [57]. For input x, the segmentation model generated a spatially corresponding mask $\hat{M}$:

$$\hat{M}=f_{\theta }\left(x\right)$$

where $f_{\theta }$ is the segmentation model and $\hat{M}$ the predicted fold-probability map. Pixel probabilities were mapped to [0, 1] using the sigmoid function:

$${\hat{p}}_{ij}=\sigma \left(z_{ij}\right)=\frac{1}{1+e^{-z_{ij}}}$$

where $z_{ij}$ is the logit at pixel (i,j) and ${\hat{p}}_{ij}$ its Fold probability. The map was binarized as:

$${\hat{M}}_{ij}=\{\begin{array}{ll} 1, & {\hat{p}}_{ij}\geq \tau \\ 0, & {\hat{p}}_{ij}<\tau \end{array}$$

where τ is the threshold; ${\hat{M}}_{ij}=1$ denotes Fold and ${\hat{M}}_{ij}=0$ background.

### 2.7. DeepLabV3-ResNet50 Architecture

DeepLabV3-ResNet50 was used for segmentation [58,59]. ResNet50 extracted image features, and DeepLabV3 produced pixel-level predictions using atrous convolution to capture wider context [60]:

$$Y\left(i,j\right)=\sum_{m}\sum_{n}X\left(i+rm,j+rn\right)K\left(m,n\right)$$

where r is the atrous ratio; r=1 is standard convolution, whereas increasing r broadens the contextual field for fold regions of varying size.

The final DeepLabV3 layer was configured for single-channel binary segmentation, yielding a fold probability for each pixel.

### 2.8. Segmentation Loss Function

Binary cross-entropy (BCE) and Dice losses were combined to address background dominance when fold regions occupied a small portion of the image [61]. BCE was defined as:

$$L_{BCE}=-\frac{1}{N}\sum_{i=1}^{N}\left[y_{i}log\left({\hat{p}}_{i}\right)+\left(1-y_{i}\right)log\left(1-{\hat{p}}_{i}\right)\right]$$

where N is the number of pixels, $y_{i}$ the ground-truth label, and ${\hat{p}}_{i}$ the predicted fold probability. Dice overlap was calculated as [62]:

$$Dice=\frac{2\left|M\cap \hat{M}\right|+\epsilon }{\left|M\right|+\left|\hat{M}\right|+\epsilon }$$

where M is the true mask, $\hat{M}$ the predicted mask, and ϵ a constant preventing division by zero. Dice loss was:

$$L_{Dice}=1-Dice$$

The total segmentation loss was:

$$L_{Seg}=L_{BCE}+L_{Dice}$$

The combined loss jointly optimized pixel-level classification and regional overlap for small and irregular fold regions.

Model parameters θ were updated by backpropagation:

$$\theta_{t+1}=\theta_{t}-\eta \nabla_{\theta }L\left(\theta_{t}\right)$$

where η is the learning rate and $\nabla_{\theta }L$ the loss gradient. AdamW was used for optimization [63], with the following regularization term:

$$L_{reg}=L+\lambda ∥\theta {∥}_{2}^{2}$$

where, λ represents the weighting attenuation coefficient. This term contributes to the model learning more generalizable representations by limiting its overfitting to the training data. For assessing the problem of false positives, the developed model was further tested on 899 images without artifacts for which the expected tissue fold mask was an empty one. The issue of false positives was assessed through the percentage of pixels classified as a fold region and also by the image-level false positive rate for which the tissue fold region was at least 1%.

### 2.9. Explainability: Visualization of Fold Foci with Grad-CAM

Grad-CAM was used to visualize regions supporting classification decisions [64]. It combines activation maps from the last convolutional layer with class-specific gradients [64,65]. For feature map k and class c, the importance weight was:

$$\alpha_{k}^{c}=\frac{1}{Z}\sum_{i}\sum_{j}\frac{\partial y^{c}}{\partial A_{ij}^{k}}$$

where $A^{k}$ is feature map k, $y^{c}$ the score for class c, and Z the number of spatial locations. The activation map was:

$$L_{Grad-CAM}^{c}=ReLU\left(\sum_{k}\alpha_{k}^{c}A^{k}\right)$$

Grad-CAM maps for the Fold class were generated to determine the areas that play a part in the classification process. These Grad-CAM maps were used only as an additional explanation and were not used as the segmentation masks; localization was performed by using a separate segmentation network [66]. The Grad-CAM maps were tested quantitatively by calculating the Dice coefficient, IoU, pointing game accuracy, and centroid distance on Grad-CAM maps created for 29 images containing folds, along with manual masks. During the pointing game test, localization was considered successful if the point of maximum Grad-CAM activation lay inside the manual tissue fold mask.

For calculating Dice and IoU, the Grad-CAM score was initially normalized into the [0, 1] interval and then binarized by keeping the top 20% of activations, that is, 80th percentile threshold in the context of that particular image. The centroid of Grad-CAM score was the weighted centroid based on activations, whereas the centroid of the manually annotated mask was found using the average coordinate of the pixels within the tissue fold mask. The Euclidean distance between both centroids was normalized by dividing it with the image diagonal length.

## 3. Results

**Figure 2:**
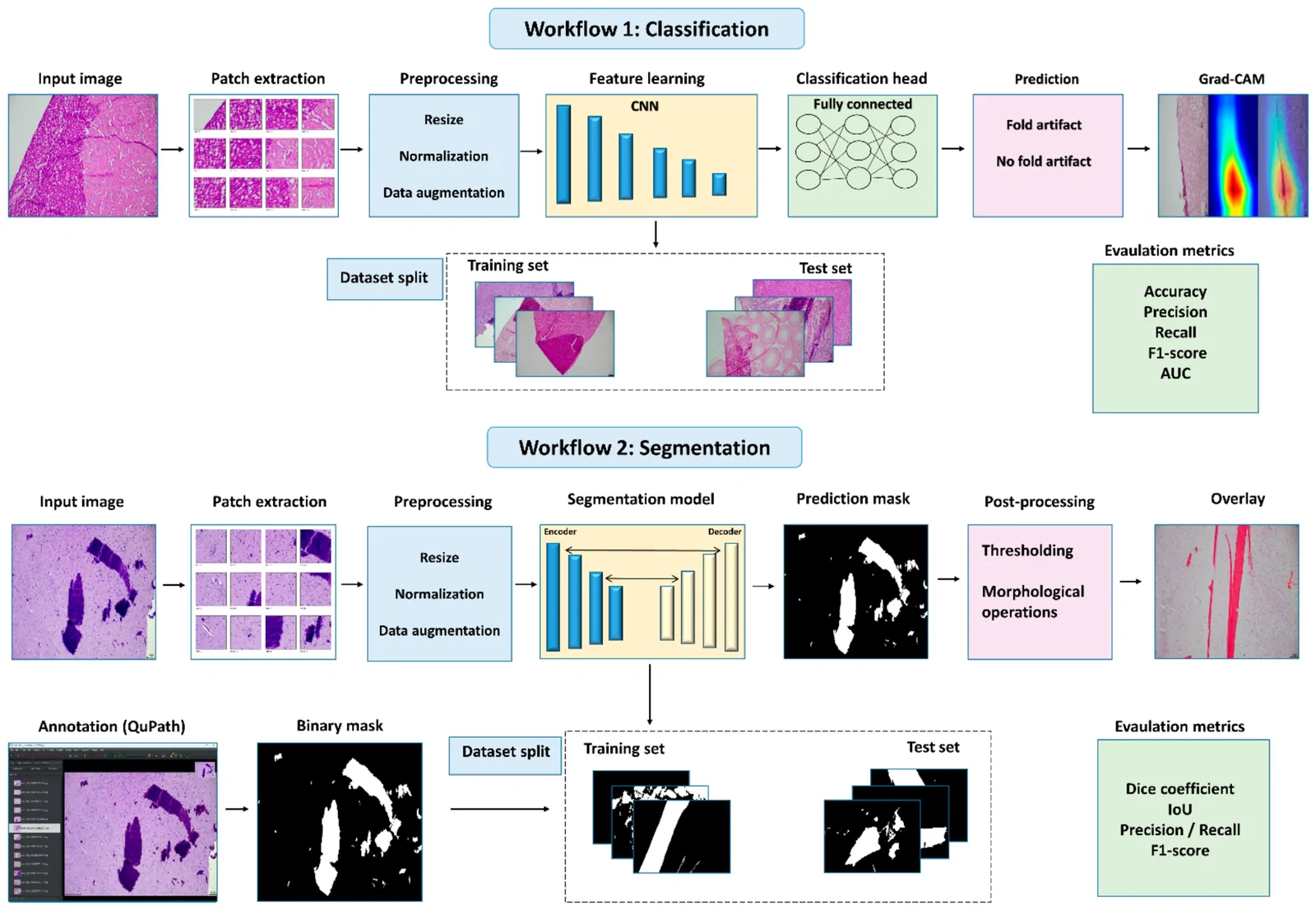
Classification and segmentation workflows for tissue fold artifacts in H&E-stained histological images.

Experimental studies were conducted on a Windows-based workstation using a Python 3.11.15-based workflow configured on the Anaconda environment. PyTorch 2.7.1 and Torchvision 0.22.1 were used for the development and training of deep learning models [67]. In image-level clean/folded classification experiments, pre-trained CNN and transformer-based models were compared; ResNet18, ResNet50, DenseNet121, EfficientNet-B0, EfficientNet-B3, ConvNeXt-Tiny, and Swin-Tiny architectures were evaluated within the same experimental framework. For the classification models, images were resized to 224 × 224 pixels, the batch size was set to 64, the learning rate and weight decay were both set to 10^−4^, and AdamW was used for optimization. In organ-independent generalization analyses, a ResNet18-based model was used, with a batch size of 32 and a learning rate of 10^−4^. A DeepLabV3-ResNet50-based semantic segmentation model was trained to determine fold regions at the pixel level; in this model, images were scaled to 512 × 512 pixels, with a batch size of 4, epoch count of 50, threshold value of 0.5, and a learning rate of 10^−4^. In all experiments, a fixed seed value of 42 was defined to reduce the effect of randomness, and the models were run on an NVIDIA GeForce RTX 3090 GPU with CUDA support. Classification performance was evaluated using accuracy, precision, recall, F1-score, AUC, and confusion matrix metrics; segmentation performance was evaluated using Dice and IoU values [68,69]. In addition, Grad-CAM based heat maps were created to visually examine the regions determined by the classification models, and the areas to which the model is sensitive were visualized on sample images of different organs. The workflows are shown in Figure 2.

### 3.1. Dataset Construction, Image Acquisition and Annotation

The present dataset has been created using histological teaching slides taken from the Department of Histology and Embryology, Faculty of Veterinary Medicine, Firat University. Consisting of 2127 H&E-stained images, the dataset comes from 302 histological training specimens taken from the brain, kidney, liver, small intestine, and testis tissues. The numbers of specimens according to organs are: brain (*n* = 51), kidney (*n* = 62), liver (*n* = 75), small intestine (*n* = 62), and testis (*n* = 52). No new animal work, sampling of tissues, or preparation of histological specimens have been performed for this research project. For the purpose of this study, H&E stained training specimens used in the practical course of histology training program of the Department of Histology and Embryology have been examined. Thus, the unit of sampling for this research is not an animal or newly taken tissue specimens, but histological preparations and images of them. Image acquisition has been done using a Nikon Eclipse Ci-L Plus microscope along with Tucsen TrueChrome 4K Pro digital camera. All images have been acquired using Mosaic 3.0 software with 10× magnification, and all these images have been stained with H&E. The analysis was performed on individual microscopic images acquired at 10× magnification rather than on whole-slide images of the entire histological slide.

The dataset was divided into two computational classes, clean and fold. The Clean category includes images that lack any tissue folding artifact in the observed image area and where the histological structure is maintained. The Fold category includes images that show tissue thickening due to tissue folds, contacts between cells and tissues, changes in staining intensity, and disruption of histological structure. A total of 899 images in the dataset have been found to be clean/artifact-free images, and there are 1228 images with tissue fold artifacts in the dataset. With respect to organs, the brain has a total of 433 images, among which 215 images are clean/artifact-free and 218 images have tissue fold artifacts. Similarly, the kidney has 466 images where 181 are clean/artifact-free and 285 images are tissue fold artifact images. Liver has 437 images, out of which 167 images are clean/artifact-free and 270 are tissue fold artifact images. The small intestine has 365 images with 152 being clean/artifact-free and 213 being tissue fold artifact images. Lastly, the testis has 426 images where 184 images are clean/artifact-free and 242 are tissue fold artifact images. All images are available in JPG file with a resolution of 3840 × 2160 pixels.

In addition to the dataset, artifact regions in histological images showing the presence of tissue fold artifacts have been manually annotated using the QuPath v0.7.0 software [70]. The annotation procedure was carried out by the first author, who is a member of the Department of Histology and Embryology, and all annotations were verified to be histologically appropriate by the second author, a specialist in Histology and Embryology. The tissue fold artifacts are considered to be technical artifacts arising due to folding of the section on itself during preparation and disturbing the normal histological structure. The basic morphological features of the regions that can be used to recognize them include an increase in tissue thickness locally, overlapping of cell and tissue structures, increased H&E stain intensity, and disruption of normal tissue architecture. At the time of annotation only those regions that show morphological alterations caused by tissue fold artifact were marked. The border between the folded tissue and normal adjacent tissue was drawn based on the analysis of tissue thickness, structural overlapping, staining intensity, and disruption of tissue architecture simultaneously. When the borders were ambiguous, annotation stopped when the histological structure returned to normal. The borders of recognized artifact regions were manually outlined, and binary masks of tissue fold artifact images were created [70,71].

### 3.2. Organ-Based Classification Performance

In this subsection, the model’s classification performance on different organ tissues was evaluated on an organ-by-organ basis. For this purpose, one organ was set aside as test data in each experiment, while images of the remaining organs were used for model training [72]. Thus, the extent to which the model could distinguish between clean and folded images of an organ tissue it had not seen during the training phase was examined. This evaluation approach aims to reveal not only the model’s success in classifying samples within the same data distribution, but also its capacity to generalize to different organ tissues [73].

**Figure 3:**
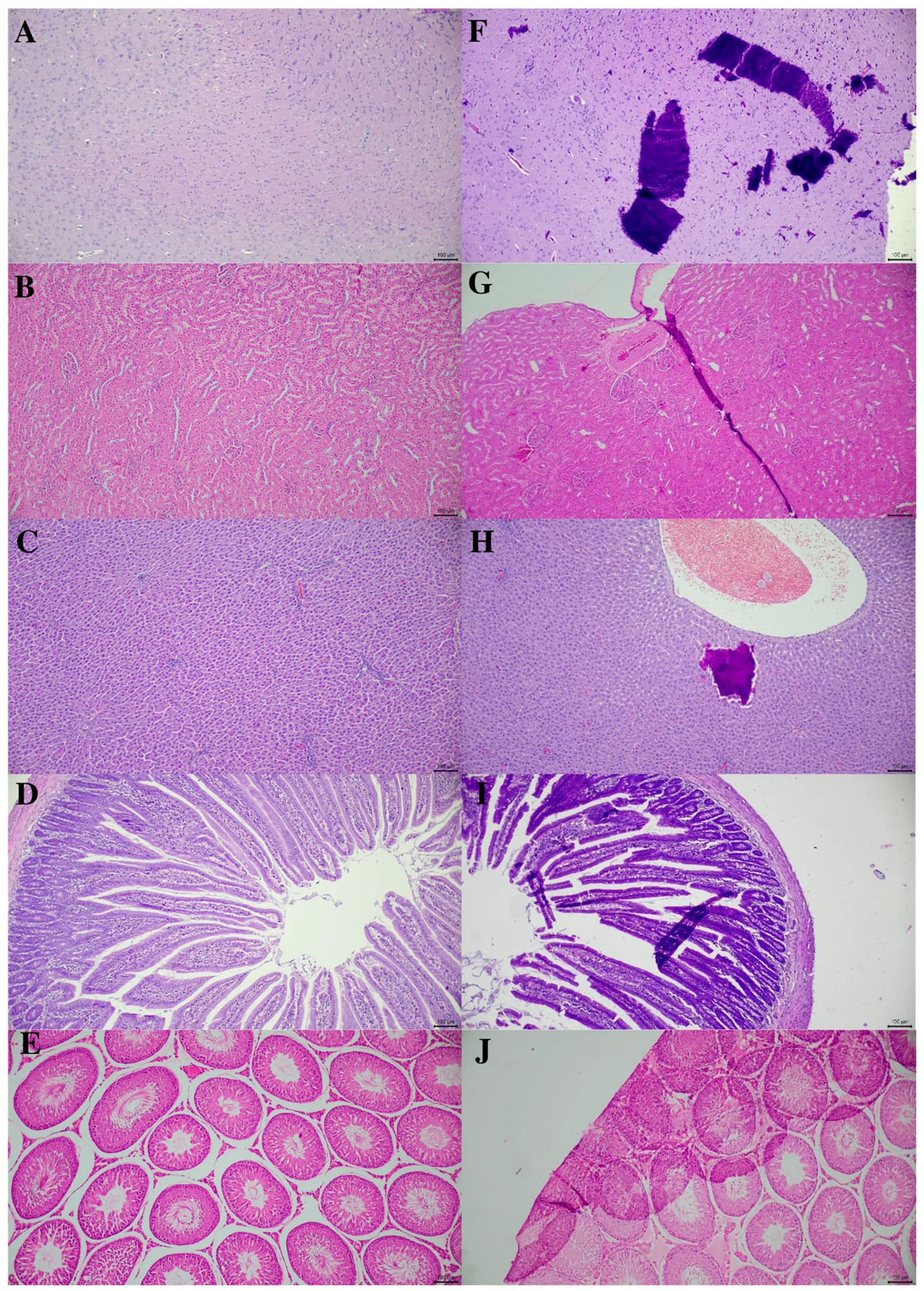
Representative H&E stained histological images of histology laboratory teaching slides. Clean/artifact-free images are presented in the left column: (**A**) brain, (**B**) kidney, (**C**) liver, (**D**) small intestine and (**E**) testis. Images with tissue fold artifacts are presented in the right column: (**F**) brain, (**G**) kidney, (**H**) liver, (**I**) small intestine and (**J**) testis. All images were obtained at 10× magnification.

Table 1 presents the organ-based classification accuracies of different deep learning models. Overall, the results show that the models exhibit high classification accuracy in the brain, kidney, liver, and testis. The fact that many models achieved accuracy values above 90% in these organs indicates that tissue fold artifacts can be largely distinguished across different tissue samples. Specifically, in the brain, the EfficientNet-B0 model achieved the highest accuracy with 98.61%, while in the kidney, Swin-Tiny stood out with 95.49%. In the liver, the highest performance was obtained from the ConvNeXt-Tiny model with 96.57%, while in the testis, the DenseNet121 and EfficientNet-B3 models yielded the most successful results with an accuracy value of 99.30%. In contrast, a significant performance decrease was observed in the small intestine across all models. For this organ, accuracy values ranged from 60.00% to 80.27%, with the highest result obtained by the EfficientNet-B3 model. This suggests that the texture, color distribution, or morphological features in the small intestine images create a visual distribution different from other organs. Therefore, the model had more difficulty distinguishing tissue fold artifacts in samples from this organ. In particular, the lack of any image of the organ tested in the training set, as discussed in terms of the leave-one-organ-out approach, suggests that the noted reduction is due to variation between organs. Figure 3 presents representative H&E-stained histological images from the five organs, including clean/artifact-free images and images containing tissue fold artifacts.

#### Error Analysis of Small-Intestine Classification

Since the small intestine had shown poor performance in the leave-one-organ-out analysis, a further error analysis was carried out to determine the cause of the errors in classification.

Table 2 presents the organ-specific classification performance for the small intestine under the leave-one-organ-out evaluation protocol. Despite the fact that the AUC metric attained quite a high value of 0.8984, the overall accuracy was equal to 0.6767. This reduction is mainly caused by an imbalance between sensitivity and specificity metrics. The sensitivity was equal to 0.9859, meaning that almost all images with the presence of tissue folds were recognized, but the specificity decreased down to 0.2434. In agreement with this trend, 115 false positives and 3 false negatives were recorded. This pattern indicates that the model was more likely to misclassify normal small-intestinal structures as tissue fold artifacts than to miss actual tissue fold artifacts. It is possible that the complexity of mucosal structure of the small intestine, which contains numerous villi, folds, and heavily stained structural elements, caused the appearance of visual patterns resembling technical tissue fold artifacts.

**Figure 4:**
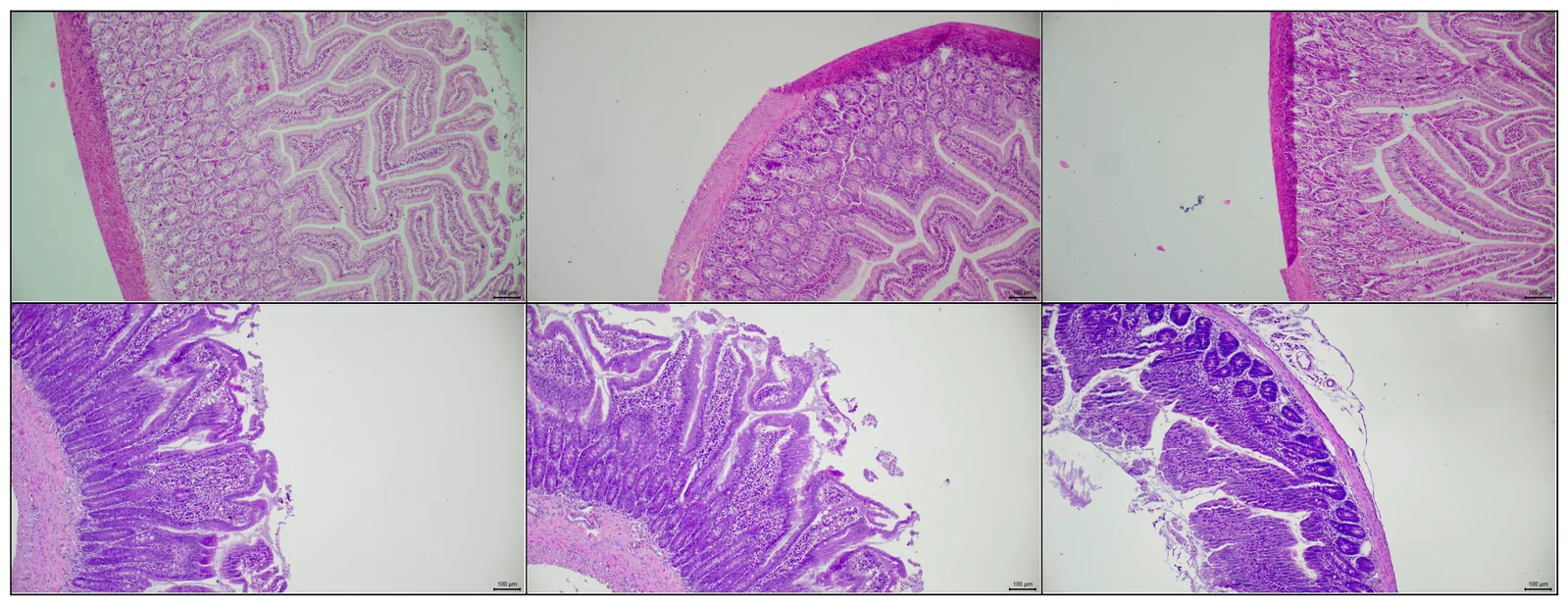
Representative small-intestine misclassifications in the leave-one-organ-out evaluation. The upper row shows false-positive cases, whereas the lower row shows false-negative cases.

Figure 4 depicts some of the representative misclassified images of the small intestine. The false-positive examples contained normal small-intestinal structures that visually resembled tissue fold artifacts. This is due to the fact that the close packing of villi, irregularities in the structure of the mucosa, variations in the plane of section, and high density of staining give rise to areas with increased structural complexity which could be confused with fold artifacts. The false negatives on the other hand showed relatively mild changes in the structure of folds with most of the tissues still retaining their structural integrity.

### 3.3. Classification Performance Using the Slide-Level Train–Test Split

In this part of the experiment, the classification accuracy was analyzed based on the train/test split performed at the level of slides. In order not to have images from the same histological slide group in both training and test sets, all the images belonging to a certain slide group were included either in the training set or in the test set.

The classification performance obtained through the train–test split at the level of slides is shown in Table 3. In order to avoid the leakage of similar microscopic fields between the training and testing data, images taken from the same histological slide groups were included into the same split. Depending on the method, the accuracy varied between 98.38% and 99.54%, the F1-scores between 98.57% and 99.59%, and the area under the receiver operating characteristic curve (AUC) between 99.79% and 99.99%. EfficientNet-B3 showed the best classification results, having accuracy of 99.54% (95% CI: 98.91–100.00%), sensitivity of 99.19%, specificity of 100.00%, F1-score of 99.59%, and AUC of 99.99%. The UNI pathology foundation model, used as a feature extractor with a linear classifier, reached accuracy of 98.61%, F1-score of 98.78%, and AUC of 99.79%.

### 3.4. Classification Performance Using the General 80/20 Data Split

In this section, the overall classification performance of the model was examined by evaluating images of all organs together. For this purpose, clean and folded images of the brain, kidney, liver, small intestine, and testis were combined into a single dataset; then, 80% of the dataset was allocated for model training and 20% for testing. This evaluation approach aims to reveal the extent to which the model can distinguish general folding patterns learned from different organ images within the same data distribution. Unlike organ-based evaluation, the test set in this stage includes samples from the organ distribution represented during the training process. Therefore, the results obtained demonstrate the overall classification capacity of the model within the dataset; rather than organ-independent generalization performance, it evaluates its overall discrimination ability on mixed images from different organs.

Table 4 presents the classification results obtained using an 80/20 training-test split with images of all organs. The results show that all evaluated models exhibited very high performance in distinguishing between clean and folded images. The highest accuracy, F1 score, and AUC values were obtained with the Swin-Tiny model; this model achieved 99.06% accuracy, a 99.19% F1 score, and a 99.98% AUC value. The EfficientNet-B3 model also produced results very close to those produced by Swin-Tiny and ranked second in terms of overall performance. The fact that the accuracy values are above 98% in all models indicates that tissue fold artifacts can be strongly distinguished within the same data distribution. However, these results reflect an evaluation structure where all organs are represented in the training and test sets.

To more reliably evaluate overall classification performance, a five-fold cross-validation approach was applied in addition to the 80/20 training-test split [74]. In this evaluation process, the dataset was divided into five subsets; in each iteration, one of these subsets was used as test data while the remaining four subsets were used for training the model. Thus, each image was tested once, and model performance was evaluated without being tied to a single data split. These metrics included accuracy, precision, recall, specificity, F1 score, Area Under the Receiver Operating Characteristic Curve (AUC), and losses, which were provided for five runs of the algorithm as means and their standard deviations [68]. Such an approach allowed measuring the consistency of model performance while discriminating between clean and folded images.

Table 5 presents the classification performance of different deep learning models according to the five-fold cross-validation test. Overall, all models demonstrate high and stable accuracy in distinguishing between clean and folded images. Accuracy values range from 98.21% to 98.73%, and F1-score values above 98% for all models indicate strong classification success not only in accuracy but also in inter-class balance. The highest accuracy and F1-score values were obtained with the ResNet50 model. However, the EfficientNet-B0, DenseNet121, ConvNeXt-Tiny, and ResNet18 models also produced very similar results, showing that different architectures can learn similar levels of distinguishing features in this task. The fact that the AUC values were above 99.8% for all models indicates that clean and folded images can be quite clearly separated in the model decision space. When test loss values were examined, the lowest loss was obtained in the DenseNet121 model, but performance differences were generally limited. The low standard deviation values indicate that the models maintained similar success levels across different folds and that the results were not dependent on a single data split.

### 3.5. Segmentation of Tissue Fold Regions

This section presents the results of segmentation aimed at identifying fold regions at the pixel level in histological images containing tissue fold artifacts. In classification experiments, the model determined whether the image was clean or folded, while in the segmentation phase, each pixel was considered as either a background or fold region. To achieve this goal, a semantic segmentation algorithm based on the DeepLabV3-ResNet50 architecture was trained on manually annotated tissue fold masks. Probability maps created by this model were binarized with the help of thresholding and compared to the ground truth masks. Segmentation results were evaluated in terms of Dice coefficient and Intersection over Union metrics. Also, the generated masks were overlaid on original images for qualitative analysis [75].

**Figure 5:**
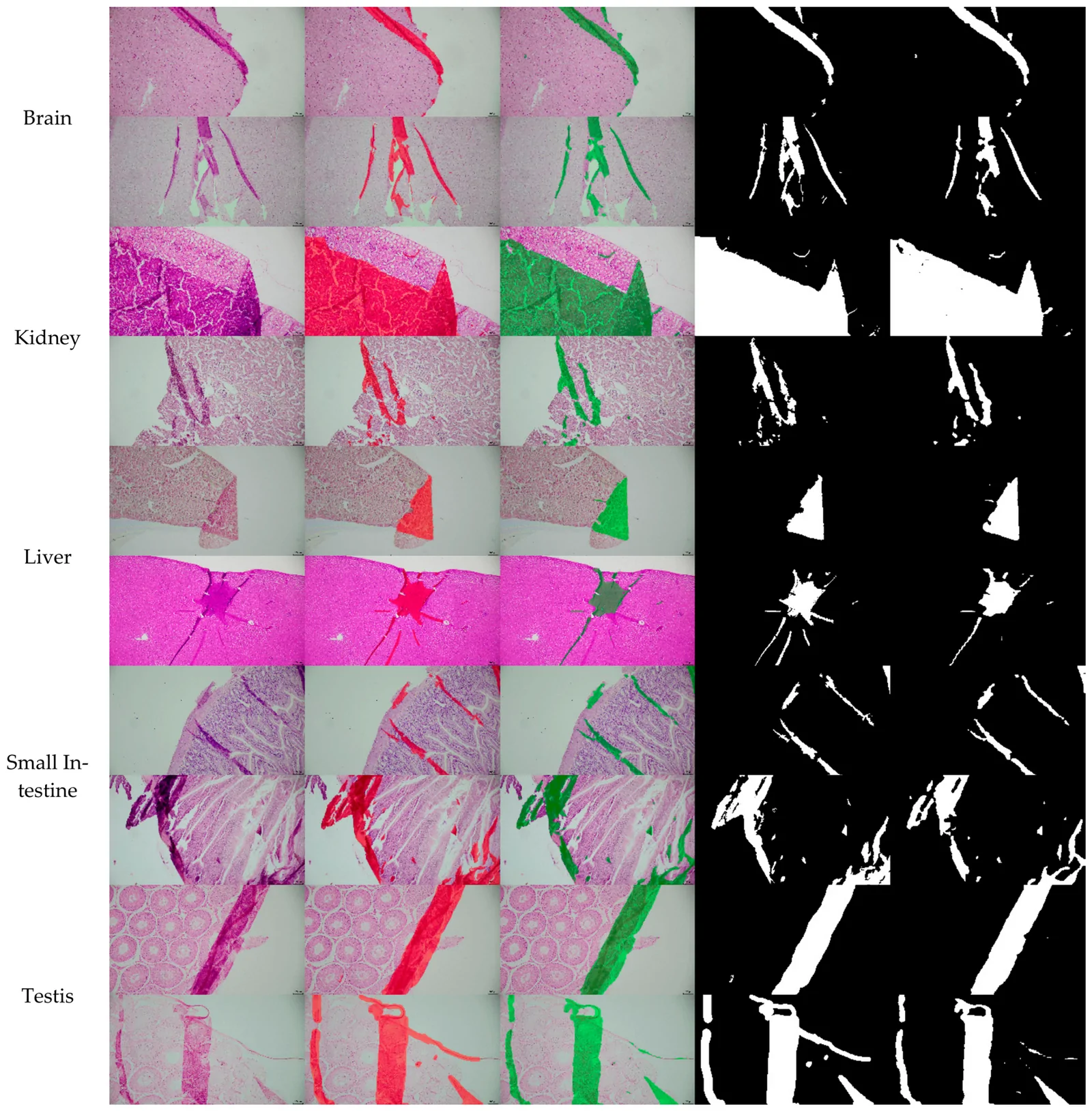
Segmentation results of the proposed approach on different organs to localize the tissue fold artifacts. In the case of each organ, the order of the columns is as follows: (i) input image, (ii) ground-truth mask, (iii) predicted segmentation mask, (iv) binary ground-truth mask, and (v) binary predicted mask.

Figure 5 shows exemplary results of segmentation for five organs with both easy and hard cases. For each example, the image is shown along with its ground truth overlay, predicted overlay, ground truth binary mask, and predicted binary mask. It is clear from these examples that the model can detect the main tissue fold regions in most of the images, although there are differences between the predicted masks and the manual masks for more challenging cases. In general, the errors in such cases are related to either partial localization of the tissue fold region or predicted additional areas which are not parts of the actual folds, especially in those cases where the borders of the folds were not clearly visible. These visual differences are consistent with the statistical differences that we observed in Table 6.

Table 6 shows the accuracy performance of the tissue fold artifact segmentation using the proposed segmentation model based on DeepLabV3-ResNet50 network in the test images of different organs in terms of the Dice and IoU metrics. In Table 6, n represents the number of test images used for each organ. In this table, the Dice value represents the similarity level of the predicted tissue fold mask and the actual mask, while the IoU value represents the intersection-over-union of the two masks [76].

Table 6 demonstrates Dice and IoU coefficients of each organ based on the final DeepLabV3-ResNet50 architecture trained using both BCE and Dice loss functions. Only images with the tissue fold artifact and their binary mask annotations are used for the segmentation task. The selected image-masks are separated into three subsets: training (70%), validation (15%), and test (15%). Accordingly, organ-based segmentation results were obtained for an independent test subset including 185 images. On this test set, the model showed the overall Dice coefficient equal to 0.7630 ± 0.2425 and the overall IoU coefficient equal to 0.6661 ± 0.2577. As follows from Table 6, the best segmentation results are achieved for the testicular tissue (Dice = 0.8291; IoU = 0.7346). In addition, relatively good results were achieved for the kidney images (Dice = 0.8168; IoU = 0.7251). The smallest overlap is shown by the small intestine images (Dice = 0.7057; IoU = 0.5897). The relatively large standard deviations for some organs, particularly for the liver, demonstrate the variance of segmentation performance per each image.

Finally, the performance of the segmentation algorithm was tested on all 899 artifact-free images in terms of occurrence of false positives. The average predicted fold-area percentage in all image pixels was 0.0234%, giving 99.9766% of pixel-level specificity. Although some minor areas of folds were found in some images, just 0.33% of artifact-free images had a fold-area percentage above 1%, with no fold-area percentage exceeding 5%. As for organs, small intestines were most prone to false positives, showing an average predicted fold-area percentage of 0.0734% (1.32% at the ≥1% level).

### 3.6. Grad-CAM Based Visual Explanation Analysis

This section uses Grad-CAM analysis to visually examine the classification model’s decisions for clean and folded histological images. Grad-CAM is a class-separation visualization method that shows which regions of the image the model utilizes more when making a specific class decision [64]. In this study, Grad-CAM was applied not for pixel-level segmentation of the tissue folding artifact, but to explain the classification model’s decision-making process. Thus, the sensitivity of the model to specific areas of the tissue when making a clean or folded decision was examined. Grad-CAM visualizations were generated on sample images of different organs. In folded images, heat maps show regions supporting the Fold class decision, while in clean images, they show regions supporting the Clean class decision. This analysis allows the model’s decision to be evaluated not only by numerical performance values but also by spatial focus areas on the image. Specifically, in folded images, it was visually examined whether the model focused on regions where tissue continuity was disrupted, color intensity changed, or folding lines became more pronounced. Grad-CAM outputs were not directly interpreted as actual tissue fold masks, because Grad-CAM is not a segmentation method that produces precise boundaries at the pixel level, but rather an explanatory technique that shows regions contributing to the classification decision [64]. Therefore, in this study, pixel-level fold localization was evaluated with a segmentation model, while the visual explainability of classification decisions was evaluated with Grad-CAM analysis.

**Figure 6:**
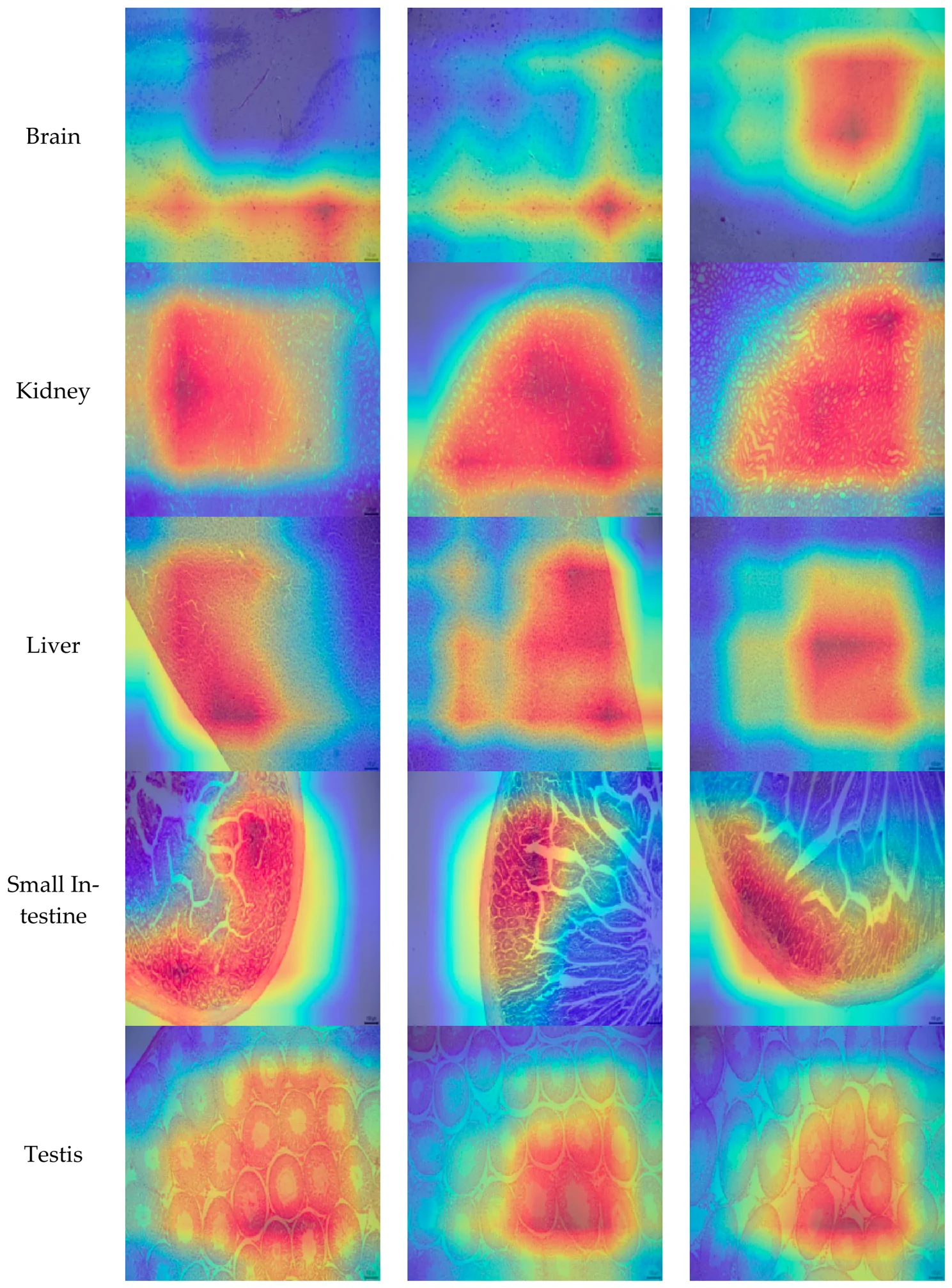
Grad-CAM overlay visualizations for clean/artifact-free histological tissue images from different organs.

Figure 6 shows Grad-CAM overlays of clean organ tissues. Examination of the images reveals that the model focuses not on a single limited region when making clean class decisions, but on broader areas reflecting the overall integrity of the tissue structure. In the brain, kidney, liver, small intestine, and testis samples, the concentration of hotspots is mostly on the morphological structure, color distribution, and continuous histological patterns of the tissue, suggesting that the model evaluates clean images based on areas without fold lines or sharp tissue distortion. This indicates that the classification model does not base its clean class decision solely on background or random image regions, but also considers the structural characteristics of the organ tissue.

**Figure 7:**
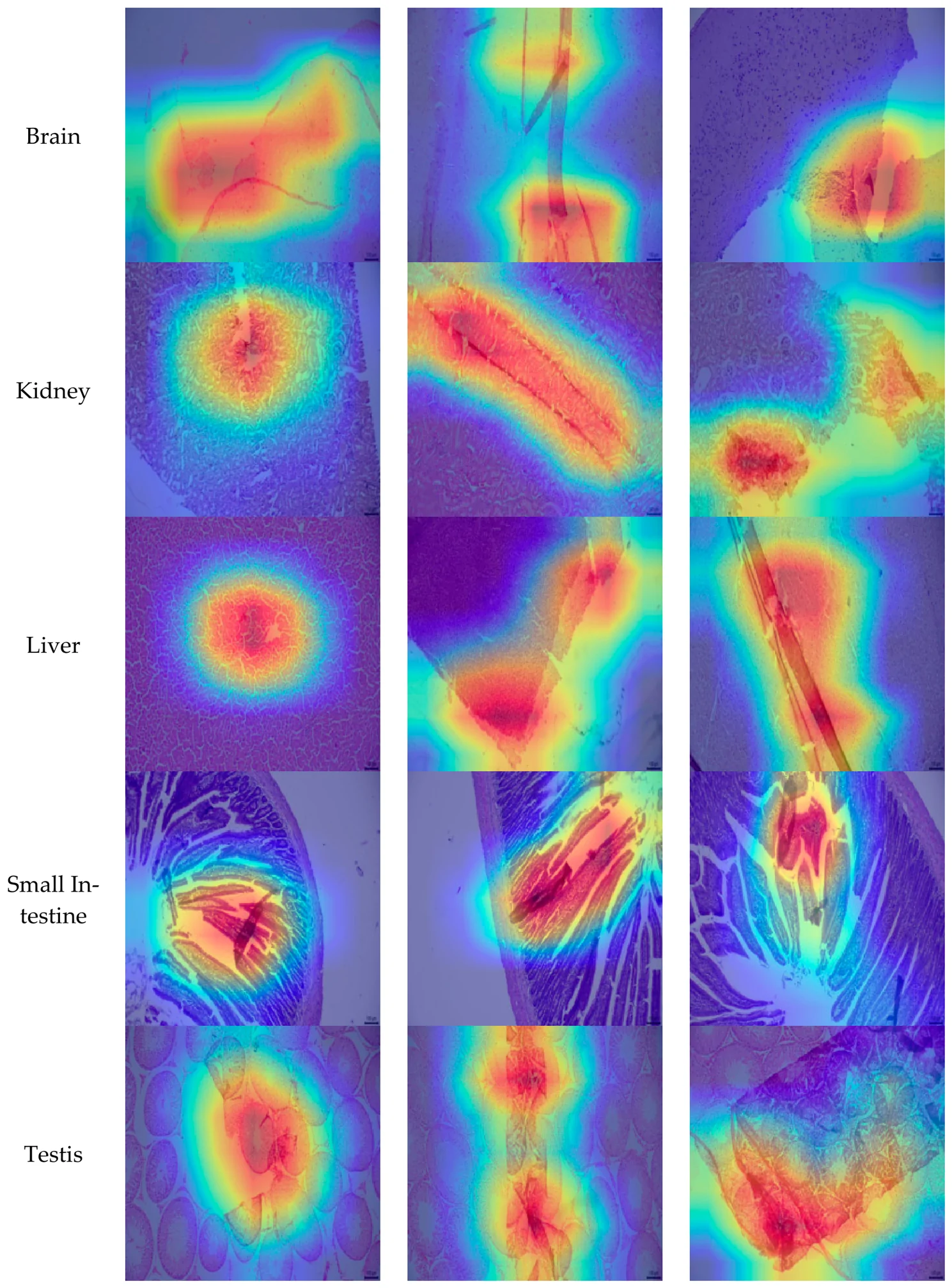
Grad-CAM overlay images for selected folded histological tissue images for different organs. Warm regions depict the parts that were most involved in the model’s decision for the Fold class.

In Figure 7, Grad-CAM overlaid images of the histological tissue images for different organs exhibiting tissue fold artifacts have been shown. The analysis of the images reveals that while making the decision for the Fold class, the model mainly focuses on the areas having folding lines, overlap of tissues, and change in color. In the brain, kidney, liver, small intestine, and testis samples, the significant overlap of hotspots with areas where the artifact is prominent indicates that the classification model bases its decision on visual patterns associated with folding, rather than random background regions. However, in some samples, the heatmaps extend to tissue regions surrounding the fold region. This is due to Grad-CAM not being a segmentation method that produces precise boundaries at the pixel level; therefore, these images should be evaluated to clarify the classification decision.

Quantitative Grad-CAM localization results are presented in Table 7. In this case, N stands for the number of fold-containing images, for which a proper correlation between the histologic image and its manually segmented tissue fold mask exists. A total of 29 images covering five organs were analyzed. As a result, we obtained Dice coefficient of 0.2423 and IoU of 0.1441. This means that the correlation between the activation area of the Grad-CAM and manually segmented fold region is quite low. However, it should be noted that certain differences were noticed in organ-specific localization behavior. The best localization performance was shown in the testis (Dice = 0.3123; IoU = 0.1911), followed by the kidney (Dice = 0.2987; IoU = 0.1828). The highest pointing-game accuracy was reached for testis and small intestine (66.67%), while there were no successful attempts to localize the maximum activation point inside the manually segmented fold region in the brain. On average, the pointing-game accuracy was equal to 41.38%. Concerning the consistency of localization results, the lowest value of the normalized centroid distance was detected for testis (0.0896), i.e., the spatial center of Grad-CAM activation correlates well with the center of manually segmented fold in this organ. The highest centroid distance was observed for brain (0.1664). All these data show that the correlation between Grad-CAM activation and manually segmented fold regions depends on the organ. Despite the lack of perfect spatial correlation, localization was more consistent for testis and, partially, for kidney and small intestine, while poor correlation was detected for brain.

### 3.7. Ablation Study

Ablation analysis was conducted to more clearly evaluate the effect of the loss function used in the segmentation phase on model performance [77,78]. Determining tissue fold artifacts at the pixel level requires not only the correct assignment of each pixel to a class, but also a high regional overlap between the predicted mask and the actual mask. Therefore, the success of the segmentation model can be directly affected by the structure of the loss function used [79]. In particular, the fact that the tissue fold region sometimes occupies a limited area within the image, and sometimes has irregular and wide boundaries, makes the choice of loss function crucial.

In this context, three different loss function configurations were compared for the DeepLabV3-ResNet50-based segmentation model. In the first experiment, only BCE Loss was used, and the model learned based on the pixel-level classification error. In the second experiment, only Dice Loss was used to directly optimize the overlap between the predicted mask and the actual mask. In the third experiment, BCE Loss and Dice Loss were used together. This combined configuration aimed to simultaneously improve both pixel-level classification accuracy and mask-level regional overlap [61]. In ablation experiments, the model architecture, data separation, input image size, training parameters, and preprocessing steps were kept constant; only the loss function was changed. The aim of this study is to evaluate the differences in performance obtained as a result of the chosen loss function. For every setup, performance evaluation was done based on Dice coefficient, IoU, and test loss. The findings justify the choice of the loss function used in the final segmentation model.

The result of the ablation study of loss functions used in the segmentation model is shown in Table 8. It can be seen that the use of BCE Loss only gives the worst Dice and IoU values. With the addition of Dice Loss, a modest numerical increase in mask overlap was observed, meaning the increase of the precision of pixel-wise tissue fold region demarcation. The best Dice and IoU values are attained by BCE + Dice Loss, which means that the combination of BCE Loss, which considers the error of pixel-wise classification, and Dice Loss, which explicitly optimizes region overlapping, improves segmentation.

Even though the test loss value is lower when using BCE Loss only, Dice and IoU are the main criteria for determining segmentation efficiency, thus the comparison of models was performed according to them. The fact that BCE + Dice Loss gives the highest values of Dice and IoU means that the combined loss function gives more reliable detection of tissue fold regions. That is why BCE + Dice Loss was chosen as the final segmentation model.

### 3.8. External Validation

In the external validation phase, the independent test dataset shared on Zenodo as part of the GrandQC study was utilized [31]. This dataset is presented at different resolution levels, and the MPP10 dataset was used in this study. The MPP10 dataset consists of images with a resolution of 1.0 µm/px and includes tile samples extracted from WSI images of four different organs: breast, colon, kidney, and prostate. Each organ folder contains images from different cases and corresponding expert annotation masks. Different quality classes are defined in the masks in the dataset, such as tissue, fold, dark spot/foreign object, pen marker, edge/air bubble, out of focus, and background [80]. Since the aim of this study was to distinguish fold artifacts from clean tissue images, the MPP10 dataset was converted to a binary classification structure. In this process, images with the fold label in the annotation masks were classified as Fold. Conversely, images without a significant tissue fold region, with preserved tissue region, and without dominant non-fold artifacts were grouped under the Clean class. Thus, images obtained from the breast, colon, kidney, and prostate organs were created as an independent clean/fold external validation dataset that the model had not previously seen during the training phase. This generated dataset was used solely for testing purposes; no retraining or fine-tuning operations were performed on the models. External validation included 9364 image tiles in total, comprising 4684 Clean tiles and 4680 Fold tiles, which originated from breast (*n* = 908), colon (*n* = 1950), kidney (*n* = 4137), and prostate (*n* = 2369) samples. Class assignment was based on the expert annotations provided by GrandQC: tiles with annotations for folds were considered to belong to the Fold class, whereas Clean tiles had no folds or any other artifacts. No training and tuning took place during the external experiment.

Table 9 presents the classification results obtained on the MPP10_datanew external validation set. Examination of the results shows that the highest overall success was achieved with the ResNet50 model. The ResNet50 model achieved 89.63% accuracy, 99.39% precision, 99.51% specificity, and 88.49% F1-score. The EfficientNet-B0 model was successful in detecting images containing tissue fold artifacts with a sensitivity of 90.00%, while the DenseNet121 model provided a more balanced result in distinguishing clean images with a specificity of 84.59%. Although the Swin-Tiny model reached a sensitivity of 90.88%, its specificity remained at 68.98%, resulting in more false positives in clean images. While the ConvNeXt-Tiny model achieved the highest sensitivity (91.73%), its specificity dropped to 58.43%, indicating that the model tended to favor the folding class.

### 3.9. Statistical Analysis

To determine whether there was statistical significance between the numerical differences observed among the classifiers, predictions from all seven models were analyzed using the common test set on the slide level (*n* = 433). The results obtained from the Cochran’s Q test show that there was a significant difference among the classification results from the different models (Q = 21.50, *p* = 0.0015). Pairwise comparisons were done using the exact McNemar test accompanied by the Holm method for correcting multiple comparisons. Even though the lowest uncorrected *p*-value was obtained for ResNet18 and DenseNet121 (*p* = 0.0039), the difference was not significant when corrected (*p* = 0.0820). All other pairwise comparisons were not significant when corrected.

Pairwise comparisons of differences in ROC-AUC were also carried out using the DeLong test with Holm correction. None of the comparisons obtained any statistically significant difference even after correction. Therefore, although there was a slight numerical difference in accuracy and AUC, none of the models is significantly better than the others in the pairwise comparison.

From Table 10, it can be observed that although the Cochran’s Q test shows a statistically significant difference between the classifiers, no single comparison done using the McNemar and the DeLong tests proves to be significant after Holm correction. Therefore, the small differences in classification accuracy should not be considered statistically superior.

## 4. Discussion

This study evaluated the detectability of tissue fold artifacts observed in H&E-stained histology teaching slides using deep learning-based classification and segmentation methods. It is known that artifacts arising during the histological preparation process can disrupt tissue architecture and hinder microscopic evaluation [7,9]. Therefore, the automatic identification of tissue fold artifacts is important for quality control, especially for preparations used in histology training. Approaches such as HistoQC and GrandQC are used for quality control in the field of digital pathology, and this study’s focus on training preparations is one of the key differences from existing studies [26,31].

In the 80/20 train–test analysis using general dataset splitting, all models showed good performance. The best results were obtained using Swin-Tiny, where the accuracy was 99.06%, F1 score was 99.19%, and AUC was 99.98%. Similarly, the other efficient model was EfficientNet-B3 model that gave an accuracy of 98.83%, F1 score 98.99%, and AUC was 99.95%. The accuracy above 98% by all models suggests that tissue fold artifacts can be successfully identified in comparison to clean tissue region under the same data distribution. This is supported by the ability of deep learning models to detect color, texture density, edge structure, and morphological distortions in histological images [36,39]. The slide-level approach provided a more stringent assessment, considering the fact that associated microscopic fields belonging to the same histological slide set were not segregated into the training and testing sets. EfficientNet-B3 showed 99.54% (95% CI, 98.91–100.00%) accuracy, 99.59% F1-score, and 99.99% AUC, while all architectural models retained accuracies greater than 98%. It is worth noting that the relatively small difference between the slide-level and generic image-level outcomes shows that the high level of internal performance was not mainly affected by the distribution of associated fields of the same slide set among training and testing sets. At the same time, as these two assessments used different data splits, the point estimates should not be considered as competing. Even though Cochran’s Q test was able to detect some overall differences between the classifiers, all the pair-wise McNemar and DeLong comparisons were no longer statistically significant following Holm adjustment. Thus, the small numerical differences between the top performers are not statistically meaningful. The supplementary evaluation based on UNI provided a pathology-specific benchmark for the classification task. Using UNI as a frozen feature extractor, the accuracy, F1-score, and area under the ROC curve (AUC) of UNI were 98.61%, 98.78%, and 99.79%, respectively, on the test set at the level of slides. The performance of UNI was close to the performance of pretrained models but not better than that of the fine-tuned model. This fact is important in light of the fact that the encoder of UNI was not trained on the current dataset; only the downstream linear classifier was trained. Thus, the findings suggest that features learned on large datasets of histopathology are transferable to the problem of detecting tissue fold artifacts [53]. Recently conducted benchmarking studies have also shown that pathology foundation models exhibit performance that is dependent on the nature of the downstream tasks and datasets and do not always outperform task-specific approaches [81].

The low standard deviations observed across the five folds indicate that classification performance was relatively stable across different data partitions. Among the selected classifiers, the most accurate model is ResNet50 with accuracy of 98.73 ± 0.54%. The F1-score metric is equal to 98.90 ± 0.47%, while AUC metric is 99.84 ± 0.10%. Notably, accuracy varies among other models with accuracy from 98.21% to 98.68%, which means that difference between folded and non-folded images is relatively stable. On the contrary, the significant drop in classification accuracy can be observed during the classification of small intestine tissue. Accuracy for such tissue varies from 60.00% to 80.27%. This reduction may be associated with the complex mucosal architecture and naturally occurring folded structures of the small intestine, which may visually resemble technical tissue folds. It can be concluded that classifier’s performance is determined by the morphological characteristics of the organs and dataset distribution [36,73]. The detailed error analysis further clarified the nature of the lower performance observed for the small intestine. Accuracy is 67.67% and the sensitivity is still high (98.59%), while the specificity is lower (24.34%). The number of 115 false positives and only three false negatives proves that the decrease in performance happened due to mislabeling of normal small intestinal structures as fold artifacts. It can be seen during the visual analysis that tightly packed villi, irregular shapes of mucosa, variation in section plane, and dense staining are common characteristics of false positive samples. On the contrary, false negatives included fold-related changes in the tissues which were less visible. Hence, the difficulty lay not in finding folds but in discriminating them from the complex mucosal structures of the small intestine.

The best-performing DeepLabV3-ResNet50 model with BCE + Dice loss in the segmentation step obtained a Dice score of 0.7630 ± 0.2425 and an IoU score of 0.6661 ± 0.2577 on the independent testing dataset comprising 185 images. The best performance in terms of Dice and IoU scores was obtained for the segmentation of testicular tissue, reaching 0.8291 and 0.7346, respectively. Kidneys were another highly segmented organ with good accuracy, while small intestines showed the least overlap of all organs. Variability between organs can be connected to differences in the size of folds, the shape of the boundary, staining contrast, and the general structure of the tissues. Namely, the presence of complex structure and irregularity of folds’ boundaries might negatively influence pixel-wise segmentation of the image. Relatively high standard deviation values, especially for liver images, suggest that segmentation results differed from sample to sample. However, taking into account the average Dice and IoU scores, it can be stated that the model achieved moderate overall agreement with the manually annotated masks, although considerable variability was observed across individual images and organs [61,79]. Thus, it is necessary not only to estimate visual quality of artifacts seen on histological images but also to analyze morphological data they contain. In the case of histomics-based approaches, the analysis of features related to the cell and tissue morphology, their organization and tissue structure greatly depends on the accuracy of image processing and segmentation. Since tissue folding changes local tissue thickness, staining intensity and relationships between structures, measurements obtained from such areas are likely to distort actual tissue features. Therefore, the identification and localization of tissue fold artifacts in advance can be considered as the quality control measure which will improve the accuracy of further histomic and computational histology studies [22].

The analysis of 899 artifact-free images allowed us to reveal more details about false-positive segmentations. The average predicted tissue fold region was equal to 0.0234% (pixel-level specificity 99.9766%). Only 0.33% of all images had the predicted tissue fold region equal to 1% or higher, none had folds larger than 5%. Despite the fact that the small intestine had the largest number of false positives, the majority of artifact-free images had no considerable folds segmented by the model. This means that the model avoided the excessive segmentation of fold regions in intact tissue, while also proving that the normal morphology of the small intestine was the most difficult feature to distinguish from the artifact.

From the ablation study results, it can be concluded that the best performance was achieved by the model with BCE + Dice Loss. In the case of using only the BCE Loss, the Dice score and IoU are 0.7319 and 0.6336 respectively, while only using Dice Loss results in 0.7588 and 0.6608, respectively. Thus, the use of a combined loss function that simultaneously considers the errors of classification at the level of individual pixels and overlap at the level of the region allows one to achieve better performance in the case of finding irregularly defined regions such as tissue fold artifacts [61].

The analysis of the Grad-CAM results shows that the classification models take into account fold lines, texture overlap, areas of strong staining, and regions where there is disruption in the continuity of morphological structures. In this way, it becomes clear that the model decisions correspond to histologically significant regions. However, it is necessary to note that the result of the Grad-CAM should not be regarded as segmentation mask; it is needed for understanding model decision making [64,66]. The quantitative analysis with manually created masks confirms the above-mentioned differences, with the Dice coefficient being equal to 0.2423, the IoU value reaching 0.1441, and pointing game accuracy being 41.38%. The localization efficiency also differs depending on organs, with better correlations in the case of testis and kidneys and poor correlation in the case of the brain. The study shows that the Grad-CAM algorithm generates a rough visualization of the regions that help to classify an image and not the exact locations of the fold.

In the analysis of the performance on external validation data, it was found that the ResNet50 model had the best overall performance on the independent MPP10 dataset. Accuracy, precision, sensitivity, specificity, and F1 score were 89.63%, 99.39%, 79.74%, 99.51%, and 88.49%, respectively. Low external validation performance compared to internal testing is understandable due to the fact that the external dataset includes images of different organs under various imaging and quality classes. It seems that the suggested approach works acceptably on the independent datasets but requires testing on large multi-center datasets [31,73]. The difference in performance between internal and external datasets emphasizes the importance of distribution shifts caused by differences in data collection. The domain shift caused by differences in tissue type, staining characteristics, and imaging conditions can affect the generality of computational histology [22].

The research includes several drawbacks. First, the data used in the research were obtained using preparations for histology training done in one center only. Second, the study focused on detecting tissue fold artifacts only, but there were no other types of artifacts such as air bubbles, knife marks, dye precipitation, out-of-focus parts, and tissue tears which were considered separately. Third, the masks for segmentation were done manually, so they had potential errors depending on the observer. Future research should involve generalizing the algorithm using images from different centers, magnifications, and several classes of artifacts. Generally, the results show that tissue fold artifacts in histology teaching slides can be detected accurately using deep learning algorithms and localized using segmentation models. From this point of view, the developed algorithm may be used as a quality control tool in histology laboratories to evaluate the quality of preparations.

## 5. Conclusions

This study aimed to automatically detect and localize tissue fold artifacts observed in H&E-stained histology teaching slides using deep learning-based methods. The results showed that tissue fold artifacts could be separated from clean tissue areas with high success. In the classification analysis performed with general data separation, the Swin-Tiny model showed the highest performance with 99.06% accuracy, 99.19% F1 score, and 99.98% AUC value. The classification performance did not change for the slide-level train–test split. EfficientNet-B3 showed an accuracy of 99.54%, an F1-score of 99.59%, and an AUC score of 99.99% with the constraint that images from the same slide are placed in only one subset. The results address the issue of high internal performance being mainly influenced by the relationship distribution in training and testing. Under the slide-level split, EfficientNet-B3 achieved the highest accuracy (99.54%) and AUC value (99.99%). However, no model demonstrated any statistical superiority over others in pairwise comparisons adjusted for multiple testing.

In five-fold cross-validation, the ResNet50 model demonstrated stable classification success with 98.73 ± 0.54% accuracy, 98.90 ± 0.47% F1 score, and 99.84 ± 0.10% AUC value. In organ-based evaluation, high performance was obtained in brain, kidney, liver, and testis tissues, while lower results were observed in small intestine tissue. This indicates that the natural morphological features of some organs can be visually confused with tissue fold artifacts. In the segmentation process, the final DeepLabV3-ResNet50 network was trained with a hybrid of BCE and Dice loss function to segment the tissues’ folds, and it scored a Dice value of 0.7630 ± 0.2425 with an IoU value of 0.6661 ± 0.2577 on the segmentation testing subset. The best segmentation result of the organs tested was testis tissue, scoring a Dice of 0.8291 and IoU of 0.7346. The thorough analysis showed that the accuracy of lower small intestine mainly depended on false positives in the detection of mucosal structures as sensitivity was still relatively high while specificity was quite low. False positive segmentation was not common in the absence of artifacts and only 0.33% exceeded the tissue fold region prediction value of 1%. The results of quantitative Grad-CAM showed poor agreement with the manually created masks, which proves that it can be used as an explanatory model.

For the external validation procedure, ResNet50 was able to achieve accuracy of 89.63%, precision of 99.39%, sensitivity of 79.74%, specificity of 99.51%, and F1-score of 88.49% on MPP10 dataset. Such results indicate partial generalizability of the method in data collected under different circumstances. Nonetheless, the noted decline in accuracy in comparison with the internal assessment indicates the need for additional validation with the use of multicentric datasets. Despite the validation of annotations by a second expert on histology, the second blind annotation was not conducted; thus, inter-rater agreement was not quantitatively measured. Thus, the presented study proves the ability to detect tissue folds in histology teaching slides by using deep learning techniques and localization by segmentation models. The suggested method could be used as a quality control instrument in histology laboratories to evaluate the quality of preparations and identify poor images.

## Figures and Tables

**Table 1:** Organ-based classification accuracies (%) of different deep learning models under the leave-one-organ-out evaluation protocol.

Model	Brain	Kidney	Liver	Small Intestine	Testis
ResNet18	96.77	94.85	94.28	67.67	97.65
ResNet50	92.38	86.27	94.05	61.37	97.42
DenseNet121	95.61	94.42	95.42	60.00	99.30
EfficientNet-B0	98.61	95.06	96.11	70.14	96.71
EfficientNet-B3	97.23	94.21	89.47	80.27	99.30
ConvNeXt-Tiny	95.15	85.62	96.57	72.05	91.55
Swin-Tiny	96.77	95.49	94.74	61.37	95.07

**Table 2:** Organ-specific classification performance in the leave-one-organ-out evaluation.

Held-Out Organ	Accuracy (95% CI)	Precision (95% CI)	Sensitivity (95% CI)	Specificity (95% CI)	F1-Score (95% CI)	AUC (95% CI)	FP	FN
Small intestine	0.6767 (0.5409–0.7819)	0.6462 (0.4720–0.7744)	0.9859 (0.9703–1.0000)	0.2434 (0.1765–0.3026)	0.7807 (0.6386–0.8673)	0.8984 (0.8534–0.9431)	115	3

**Table 3:** Classification performance of the evaluated models using the slide-level train–test split.

Model	Accuracy (95% CI)	Precision (95% CI)	Sensitivity (95% CI)	Specificity (95% CI)	F1-Score (95% CI)	AUC (95% CI)
ResNet18	0.9908 (0.9794–1.0000)	0.9959 (0.9833–1.0000)	0.9879 (0.9707–1.0000)	0.9946 (0.9800–1.0000)	0.9919 (0.9812–1.0000)	0.9997 (0.9989–1.0000)
ResNet50	0.9838 (0.9669–0.9976)	0.9959 (0.9832–1.0000)	0.9757 (0.9488–1.0000)	0.9946 (0.9800–1.0000)	0.9857 (0.9696–0.9975)	0.9995 (0.9987–1.0000)
DenseNet121	0.9908 (0.9809–0.9978)	0.9959 (0.9833–1.0000)	0.9879 (0.9773–1.0000)	0.9946 (0.9770–1.0000)	0.9919 (0.9838–0.9980)	0.9998 (0.9993–1.0000)
EfficientNet-B0	0.9908 (0.9785–1.0000)	0.9959 (0.9834–1.0000)	0.9879 (0.9707–1.0000)	0.9946 (0.9770–1.0000)	0.9919 (0.9811–1.0000)	0.9993 (0.9979–1.0000)
EfficientNet-B3	0.9954 (0.9891–1.0000)	1.0000 (1.0000–1.0000)	0.9919 (0.9819–1.0000)	1.0000 (1.0000–1.0000)	0.9959 (0.9909–1.0000)	0.9999 (0.9997–1.0000)
ConvNeXt-Tiny	0.9885 (0.9771–0.9977)	0.9959 (0.9844–1.0000)	0.9838 (0.9673–1.0000)	0.9946 (0.9810–1.0000)	0.9898 (0.9801–0.9978)	0.9994 (0.9984–1.0000)
Swin-Tiny	0.9908 (0.9798–1.0000)	1.0000 (1.0000–1.0000)	0.9838 (0.9663–1.0000)	1.0000 (1.0000–1.0000)	0.9918 (0.9829–1.0000)	0.9994 (0.9983–1.0000)
UNI (frozen feature extractor + linear probe)	0.9861 (0.9740–0.9953)	0.9959 (0.9833–1.0000)	0.9798 (0.9627–0.9926)	0.9946 (0.9770–1.0000)	0.9878 (0.9764–0.9952)	0.9979 (0.9948–0.9999)

**Table 4:** Classification performance of different deep learning models using the 80/20 train–test split.

Model	Accuracy (%)	F1-Score (%)	AUC (%)	Test Loss
Swin-Tiny	99.06	99.19	99.98	0.0197
EfficientNet-B3	98.83	98.99	99.95	0.0272
ResNet50	98.59	98.77	99.94	0.0383
ResNet18	98.59	98.77	99.91	0.0483
EfficientNet-B0	98.59	98.77	99.89	0.0462
DenseNet121	98.12	98.35	99.94	0.0488
ConvNeXt-Tiny	98.12	98.38	99.93	0.0343

**Table 5:** Classification performance of different deep learning models using the five-fold cross validation test.

Model	Accuracy (%)	F1-Score (%)	AUC (%)	Test Loss
Swin-Tiny	98.21 ± 0.79	98.45 ± 0.68	99.87 ± 0.09	0.0505 ± 0.0248
EfficientNet-B3	98.35 ± 0.58	98.57 ± 0.50	99.81 ± 0.10	0.0460 ± 0.0096
ResNet50	98.73 ± 0.54	98.90 ± 0.47	99.84 ± 0.10	0.0420 ± 0.0153
ResNet18	98.54 ± 0.59	98.73 ± 0.51	99.88 ± 0.06	0.0470 ± 0.0107
EfficientNet-B0	98.68 ± 0.64	98.86 ± 0.56	99.83 ± 0.14	0.0456 ± 0.0189
DenseNet121	98.64 ± 0.35	98.81 ± 0.31	99.86 ± 0.14	0.0400 ± 0.0145
ConvNeXt-Tiny	98.59 ± 0.60	98.77 ± 0.53	99.86 ± 0.08	0.0492 ± 0.0231

**Table 6:** Organ-based segmentation performance of the final DeepLabV3-ResNet50 model trained with the combined BCE + Dice loss.

Organ	n	Dice Mean	Dice Std	IoU Mean	IoU Std
Brain	32	0.7267	0.2468	0.6167	0.2506
Kidney	43	0.8168	0.1853	0.7251	0.2292
Liver	41	0.7199	0.3244	0.6405	0.3200
Small Intestine	32	0.7057	0.2423	0.5897	0.2501
Testis	37	0.8291	0.1623	0.7346	0.1984
Overall	185	0.7630	0.2425	0.6661	0.2577

**Table 7:** Quantitative evaluation of Grad-CAM localization against manually annotated fold regions across organs.

Organ	N	Dice Score	IoU	Pointing-Game Acc. (%)	Normalized Centroid Distance
Brain	5	0.1275	0.0703	0.0	0.1664
Kidney	6	0.2987	0.1828	16.67	0.1127
Liver	6	0.2176	0.1271	50.0	0.1259
Small Intestine	6	0.2361	0.1366	66.67	0.1141
Testis	6	0.3123	0.1911	66.67	0.0896
Overall	29	0.2423	0.1441	41.38	0.1202

**Table 8:** Ablation study results for different loss functions in the segmentation model.

Loss Function	Dice Mean ± Std	IoU Mean ± Std	Test Loss
BCE Loss	0.7319 ± 0.2678	0.6336 ± 0.2745	0.0726
Dice Loss	0.7588 ± 0.2422	0.6608 ± 0.2593	0.2423
BCE + Dice Loss	0.7630 ± 0.2425	0.6661 ± 0.2577	0.3250

**Table 9:** Classification performance on the MPP10_datanew external validation set (*n* = 9364).

Model	Accuracy (%)	Precision (%)	Sensitivity (%)	Specificity (%)	F1-Score (%)
ResNet50	89.63	99.39	79.74	99.51	88.49
EfficientNet-B0	82.30	77.99	90.00	74.62	83.56
DenseNet121	82.10	83.77	79.62	84.59	81.64
Swin-Tiny	79.92	74.54	90.88	68.98	81.90
ResNet18	77.64	80.73	72.59	82.69	76.44
ConvNeXt-Tiny	75.07	68.80	91.73	58.43	78.63
EfficientNet-B3	74.86	69.14	89.76	59.97	78.11

**Table 10:** Statistical comparison of the classification models on the slide-level test set.

Model Comparison	McNemar *p*	Holm-Adjusted *p*	DeLong *p*	Holm-Adjusted *p*
ResNet18 vs. ResNet50	0.3438	1.000	0.2131	1.000
ResNet18 vs. DenseNet121	0.0039	0.0820	0.1628	1.000
ResNet18 vs. EfficientNet-B0	0.0215	0.4297	0.3824	1.000
ResNet18 vs. EfficientNet-B3	0.0215	0.4297	0.0612	1.000
ResNet18 vs. ConvNeXt-Tiny	0.0215	0.4297	0.4659	1.000
ResNet18 vs. Swin-Tiny	0.0654	1.000	0.1517	1.000
ResNet50 vs. DenseNet121	0.0625	1.000	0.6552	1.000
ResNet50 vs. EfficientNet-B0	0.1250	1.000	0.8478	1.000
ResNet50 vs. EfficientNet-B3	0.2891	1.000	0.1681	1.000
ResNet50 vs. ConvNeXt-Tiny	0.1250	1.000	0.5016	1.000
ResNet50 vs. Swin-Tiny	0.4531	1.000	0.6139	1.000
DenseNet121 vs. EfficientNet-B0	1.000	1.000	0.3146	1.000
DenseNet121 vs. EfficientNet-B3	1.000	1.000	0.3149	1.000
DenseNet121 vs. ConvNeXt-Tiny	1.000	1.000	0.2785	1.000
DenseNet121 vs. Swin-Tiny	0.5000	1.000	0.9318	1.000
EfficientNet-B0 vs. EfficientNet-B3	1.000	1.000	0.2666	1.000
EfficientNet-B0 vs. ConvNeXt-Tiny	1.000	1.000	0.7344	1.000
EfficientNet-B0 vs. Swin-Tiny	1.000	1.000	0.5802	1.000
EfficientNet-B3 vs. ConvNeXt-Tiny	1.000	1.000	0.1269	1.000
EfficientNet-B3 vs. Swin-Tiny	1.000	1.000	0.1608	1.000
ConvNeXt-Tiny vs. Swin-Tiny	1.000	1.000	0.2265	1.000

## Data Availability

The dataset generated during this study is publicly available in the Zenodo repository at https://doi.org/10.5281/zenodo.21493260.
